# A study of blockchain-based liquidity cross-chain model

**DOI:** 10.1371/journal.pone.0302145

**Published:** 2024-06-11

**Authors:** Yutong Han, Chundong Wang, Huaibin Wang, Yi Yang, Xi Wang

**Affiliations:** 1 Tianjin Key Laboratory of Intelligence Computing and Novel Software Technology, Tianjin University of Technology, Tianjin, PR China; 2 Key Laboratory of Computer Vision and System, Ministry of Education, Tianjin University of Technology, Tianjin, PR China; 3 School of Computer Science and Engineering, Tianjin University of Technology, Tianjin, China; Jazan University, SAUDI ARABIA

## Abstract

Blockchain cross-chaining is about interconnectivity and interoperability between chains and involves both physical to virtual digital aspects and cross-chaining between digital networks. During the process, the liquidity transfer of information or assets can increase the use of items with other chains, so it is worth noting that the enhancement of cross-chain liquidity is of great practical importance to cross-chain technology. In this model, Layerzero is used as the primary secure cross-chain facility to build a full-chain identity by unifying NFT-distributed autonomous cross-chain identity IDs; applying super-contract pairs to enhance cross-chain liquidity; and initiating a dynamic transaction node creditworthiness model to increase the security of the cross-chain model and its risk management. Finally, by verifying three important property metrics timeliness is improved by at least 18%, robustness is increased by at least 50.9%, and radius of convergence is reduced by at least 25%. It is verified that the liquidity cross-chain model can eliminate the authentication transition between hierarchies while saving the cross-chain time cost, as a way to truly realize the liquid interoperability between multiple chains of blockchain.

## Introduction

### Motivation

With the rapid breakthrough and development of the underlying technology of blockchain, more and more enterprises actively combine their business needs to accelerate their integration into the blockchain ecology and gradually start the landing and application in cross-border payment, supply chain finance, e-healthcare, Internet of Things, anti-counterfeit traceability and other scenarios [1–4]. Blockchain is a new application model of distributed data storage, peer-to-peer transmission, consensus mechanism, cryptographic algorithms, and other computer technologies, and the original ecology with a single chain as the core has been transformed into an ecology with multiple chains co-existing. In the multi-chain ecology, cross-chain interaction has become a very important topic, and in the process of exploration, cross-chain approaches with different principles have been generated. Blockchain cross-chain development is divided into five phases, with the first phase used to enable the most basic cross-chain communication and inter-chain token movement. The second stage is used to enable users to provide liquidity to assets on different chains, thereby maximizing returns. The third stage is used to enable inter-application communication between different chains, i.e. depositing collateral on one chain while lending on another chain. The fourth phase deploys different parts of a single application on multiple chains so that each part runs on the most efficient chain and the back-end packages of these different chains will communicate with each other to ensure continuity of user experience. The fifth phase is used to provide interfaces to the broad Web3 ecosystem. The main mainstream cross-chain technologies are Notaries Schemes, Sidechain Schemes, Relay Chain Schemes, and Hash-Locking Schemes. Notaries Scheme, Sidechain/Relay Chain, Hash-Locking, and Distributed Private Key Control are the current popular cross-blockchain architecture schemes [5]. In the research and development of blockchain technology, inter-chain interoperability greatly limits the application space of blockchain. For IoT systems that apply multi-licensed chain architecture, cross-chain technology is a bridge for the single chain to expand and connect outward and is the key to realizing inter-chain value interconnection. Cross-chain technology can act between public and private chains, or between multiple private chains, and likewise between multiple sub-blockchains within a federated chain [6–8].

The existence of liquidity cross-chain technology is based on the core assumption of the emergence of a multi-chain ecological landscape in the future, with the significance of facilitating the flow of assets as well as information across different blockchains [9–11]. This cross-chain approach creates the target chain as a side chain of the source chain by deploying the smart contract of the source chain in the target chain, enabling the two chains to pass information. It would be called liquidity because such project parties using a cross-chain approach would create liquidity pools on these different chains, enabling users to exchange assets on different chains directly through these pools. This approach of relying on a unified liquidity pool to complete cross-chains significantly reduces the risk users face when encapsulating assets with other cross-chains and also increases the speed of cross-chains by eliminating the encapsulation process, as well as providing tokens as liquidity in exchange for a portion of cross-chains transfer fees or APY revenue.

### Design goals and security requirements

Each blockchain has its own communication protocol, consensus rules, governance model, and native assets [12–15]. The core of cross-chain lies in reaching consensus, allowing one blockchain to access the state of another blockchain, and promoting information and assets that can be passed between blockchains, but the liquidity cross-chain model is limited by security, interoperability, and decentralization. Among the dimensions, security is the most important, mainly due to the vulnerability of smart contracts, and secondly due to the fact that more than half of the verification nodes and private keys are obtained when using the multi-signature technology for external verification. The design metrics and main security requirements of the liquidity cross-chain model are as follows.

- Unified Distributed Autonomous Cross-Chain Identity
- Applicability of cross-chain protocols
- Security of cross-chain data and cross-chain protocols
- Mobility displaces cross-chain velocity
- Privacy Protection

### Research contributions

To sum up, the main application of liquidity cross-chain technology as a bridge connecting various blockchains is to realize the functions of atomic free trading of assets, free interoperability of information, and complementary services between different blockchains. Currently, there are limitations because blockchain services in different domains are not deployed on the same underlying blockchain infrastructure, and existing projects and technologies still lack the ability to freely communicate with each other between these infrastructures. Based on the existing research, this paper proposes a liquid cross-chain model architecture based on a “Super contract pair” with Layerzero as the basic secure cross-chain facility and investigates the construction of a unified distributed autonomous cross-chain identity NFT ID, specific contributions are listed below.

(1) For the characteristics of liquidity cross-chain, unified distributed autonomous cross-chain NFT identity ID, NFT data token in the Ethernet and other blockchain networks to transfer data and value for storage and transmission, by generating NFT ID easier access to cross-chain service framework.
(2) Based on the Layerzero service framework, ERC’s super contract is designed to manage the bonding curve of the model with AMM, so as to achieve the effect of free liquidity transfer of digital assets and free interoperability of information to achieve instant liquidity.
(3) Design a dynamic transaction node evaluation creditworthiness model to ensure the processing efficiency of transaction nodes and increase the security of the cross-chain model and its risk management.

### Paper structure

The rest of the manuscript is organized as follows: The Related work section summarizes the existing cross-chaining literature for representative cross-chaining, presents the existing problems of cross-chaining models, and finally presents the proposed liquidity cross-chaining model in this paper. The Method section provides a proposed new framework for liquid cross-chain models, which unifies the NFT distributed autonomous cross-chain ID to build chain-wide identification, designs a “super contract pair” model to enhance cross-chain liquidity, and finally initiates a dynamic transaction node creditworthiness model to increase the security of the cross-chain model and manage its risk. The Experiment Analysis and Result section presents the experimental results of the proposed model. Finally, the Conclusion section summarizes the work of this paper and provides a brief description and analysis of the limitations of the work as well as directions for future work.

## Related work

The technical model for mobile cross-chain interaction can be either a notary model or an information lock model. The so-called notary model refers to the existence of a trusted notary node, and this node has the functions and powers of multiple-chain packing and sorting, in-chain blocking, etc. Both parties across chains submit their information to the notary, and in some cases, they need to transfer assets and other information to the notary for verification, and the notary executes the exchange contract to exchange ownership, transfer exchange, and destroy/generate the information. This model is centralized, and the performance, security, and availability are completely dependent on the notary node. The so-called information lock model means that the initiator uses a puzzle and the answer to lock the information and assets to be exchanged, specifying the recipient and restrictions such as time and block height. Within the restriction, the receiver can use the answer to extract the ownership of the information, assets, etc. at any time. If they are not extracted when the restriction is reached, the information and assets are returned to the initiator. Both parties involved in cross-chain can use this technology to complete information cross-chain [16, 17]. Table 1 demonstrates the nomenclature set of this paper.

**Table 1:** Nomenclature set.

Short Title	Full Name
APY	Annualized Percentage Yield
ERC	Ethereum Request for Comment
NFT	Non-Fungible Token
AMM	Auto Market Maker
CA	Consensus Agreement
SC	Smart Contracts
TP	Trading Programs
PS	Privacy and Security
IBC	Inter-Blockchain Communication
SKD	Software Development Kit
IPFS	Inter Planetary File System
API	Application Programming Interface

Blockchain cross-chain communication can allow the exchange of information assets between blockchains. Consensus in the context of cross-chain communication is reflected in how participants from one blockchain can be sure of the state of a remote blockchain. Table 2 compares the implementation methods of the current and proposed models as well as the target strategies. Fengting Luo et al. [18] proposed an IoT many-to-many cross-domain authentication scheme based on a hybrid blockchain architecture, which enables multiple devices to simultaneously perform mutual authentication with multiple data service providers from other systems. Dalila Ressi et al. [19] summarized the potential of AI to enhance blockchain and improve the efficiency, security, and reliability of blockchain-based applications. Peter Robinson [20] analyzes how each protocol achieves cross-chain consensus, what trust assumptions are made, their ability to operate successfully in the context of permissionless and permissioned blockchains, and whether the protocols provide atomic updates across blockchains. Lydia Negka [21] et al. propose a state channel design that is optimal for applications where the state includes a large set of elements by running an RSA accumulator on a compact state structure. This state channel design is presented by analyzing all the state channel operations and how these operations are modified, and finally discusses the security of the design, while analyzing a practical usage scenario on the design of an on-chain asset (e.g., NFTs) exchange application on this basis. Xiaodi Wang [22] et al. designed a near real-time bilateral trading scheme to negotiate directly on a peer-to-peer basis without any intermediary in order to achieve a balance between supply and demand within the limits of the power network. Shahbaz Siddiqui [23] et al. propose the use of smart contracts in a multi-chain blockchain to achieve data security during collaborative tasks in smart city municipal architecture. The proposed security solution is based on the dynamic nature of smart contracts to securely govern and control all interactions and transactions between different heterogeneous IT networks. Nannan Wu [24] et al. propose an attribute-based access control scheme that benefits from smart contract technology while ensuring the privacy of attributes and policies. The scheme uses multiple blockchain nodes to collectively decrypt data and uses zero-knowledge proof techniques to ensure the correctness of the decryption results, thus ensuring that smart contracts can make authorization decisions without seeing the actual attributes or policies. Yuxian Li [25] et al. proposed ZeroCross, a novel sidechain-based privacy protection scheme that guarantees the unlinkability of cross-chain transactions, the fairness of exchanges, and the confidentiality of values. He Y [26] et al. proposed a cross-chain trusted smart contract (C2T smart contract) to ensure the authenticity of cross-chain information, real-time and inter-chain write mutual exclusion, which makes the reputation calculation in the multi-chain billing model more convenient and accurate. Pang Y [27] proposed a new consensus protocol, Multi-Token Proof of Stake (MPoS). the MPoS protocol can enhance the network effect of tokens in cross-chain ecosystems, and enable the user base of blockchain systems to grow dramatically.

**Table 2:** Comparison of current and proposed models.

References	CA	SC	TP	PS	Liquidity
[18]		✓			
[20]			✓		
[21]		✓		✓	
[22]		✓		✓	
[23]		✓		✓	
[24]	✓				
[25]			✓	✓	
[26]	✓				
[27]				✓	
Liquidity cross-chain model		✓		✓	✓

In the existing liquidity cross-chain technology, researchers can only enhance the problems related to liquidity cross-chain using a single optimization technique, while a free liquidity blockchain cross-chain model framework with simple operation, high security, and fast transaction speed is missing. In summary, the enhancement of liquidity cross-chain technology can be divided into four points as follows.

### The underlying layer is more lightweight

The IBC protocol is touted as the gold standard for cross-chaining, but there may be some practical difficulties in implementing heterogeneous chains. The first is the transition from repeaters to propagators. The main task of the repeater is to take the observed source chain information and commit it to the target chain, i.e., to complete the process of information transfer. Then, this process can be realized by means of a prophecy machine. That is, the prophecy machine changes from “down-chain to up-chain” to “up-chain to up-chain” information transfer. Secondly, the transition from the prophecy machine to TEE is to reduce the trust dependency on the prophecy machine, among which other technical means can be used to improve the work of blockhead synchronization. Finally, in the transition from Merkle Proof to ZK Proof, the current cross-chain implementation generally utilizes Merkle proof, but by generating zero-knowledge proof, it can also be used to solve the problem of the difficulty of the cost of signing Ether, as a way to avoid the high-cost calculation associated with the execution of the signature in the smart contract.

### Better development support

From generic messages across chains to smart contract calls or from better multi-chain to full-chain SDKs.

### Innovation of liquidity in the application layer

Shared liquidity AMM, based on cross-chain protocols enables management functions such as liquidity movement and settlement across chains.

### Improvements to consensus protocols

Application chains are inadequate for maintaining their own security and require some cross-chain authentication to ensure mesh security.

In this paper, we design a liquidity cross-chain model based on “super contract pairs”, which breaks through the previous fixed cross-chain model structure, adds super contract pairs with the liquidity AMM model, and unifies the whole-chain NFT distributed autonomous identity to enhance the liquidity function of the cross-chain model. At the same time, the dynamic transaction node credit model is activated to increase the security and risk management of the original Layerzero cross-chain model.

## Method

**Fig 1:**
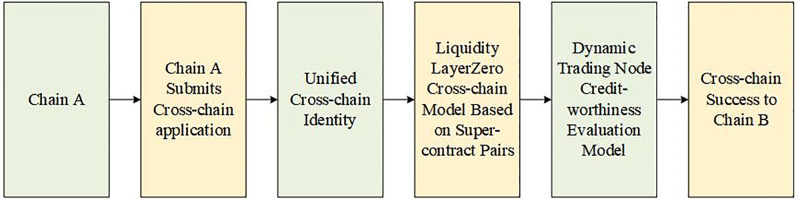
The liquidity cross-chain flow chart.

Based on the current Layerzero liquidity replacement cross-chain technology, there are problems of not being able to realize the governance model and not being able to adjust the cross-chain interaction scenarios independently. This paper introduces the concept of liquidity super contract pairs, composed of super transaction contracts and super object contracts deployed via a protocol. A super contract pair consisting of ERC transaction contracts and ERC super object contracts is deployed via protocol to create “fungible passes” to realize the cross-chain liquidity of blockchain. Finally, we design a blockchain liquidity replacement cross-chain “super contract pair” dynamic transaction creditworthiness governance model and give a specific implementation method. The liquidity cross-chain flow chart is shown in Fig 1.

### Unified cross-chain identity

In this section, the pre-processing for blockchain data unified cross-chain identity is to transform the data into NFT. NFT is a unique cryptocurrency token used to represent a digital asset, which can be understood as a certificate of ownership of a virtual or physical asset. The main difference between NFT and other cryptocurrencies such as Bitcoin is that NFT is not interchangeable with the same NFT. Blockchains based on NFT technology are tokenized with pointing data information or digital objects, and in most cases, these “things” exist in a decentralized storage layer. This makes them resistant to single points of failure and censorship, and NFTs can simultaneously give users ownership and sovereignty over their digital assets.

Data on the traditional blockchain chain is broadly classified into six categories, configuration data, account data, block data, transaction data, entity data, and contract data. Data not suitable for on-chain is divided into large files, confidential or sensitive data, and large amounts of redundant and repetitive data.

#### Production of NFT ID

NFT is a file format (commonly used to transfer messages or values over the network) and NFT exists on the blockchain, so these tokens (or files) contain properties similar to Bitcoin, mainly digital ownership (tokens in a wallet) and transparency (all activity is recorded on the blockchain). Each NFT in the blockchain has a unique token (or message), a common example of which might be a digital trading card or a digital artwork. The value of NFT will vary depending on how it is used. Currently, NFT has the following characteristics: uniqueness, permanence, programmability, no privileges, and digital ownership. The steps for generating NFT id data are as follows.

$$\begin{array}{c} id=F(I) \end{array}$$ (1)

I stand for the input substance; F stands for the processing function, which can also be called a processing method; and id stands for the unique signature intermediate product.

Step 1: Obtain data information media to determine the content of the NFT data. the NFT can support a range of files (JPG, PNG, GIF, MP3, etc.).
Step 2: Set up a wallet. For storing cryptocurrencies for rewarding receipts or penalizing payments in cross-chain transactions.
Step 3: Edit the data information on each NFT to generate NFT data.

#### Pass-through of NFT

Pass-through is the conversion of an asset into a digital pass-through on a blockchain system. The biggest difference between pass-through and securitization is the introduction of programmability into pass-through assets. By the same token, pass-through cation allows for the introduction of business logic and reduces the need for manual settlement, while smart contracts in turn have automated trading, calculation of asset prices, and other specific functions.

Pass-Through contains three key principles. a) Liquidity. Increased liquidity helps to unlock value for the market through a liquidity premium. b) Tokens with programmability. Programmability refers to the ability to introduce specific business logic into a smart contract, allowing it to automate operations. c) Immutable proof of ownership. Blockchain is immutable and can be publicly tracked for every transfer and owner. Digitally tracking transactions not only provides a history of ownership changes but also reduces fraud.

#### Smart contract of NFT

NFT smart contracts can be developed based on different public chains, and it is not limited to any one public chain. Different public chains have different implementations of smart contract schemes, and this article shows it with the Ethernet public chain. On top of Ethernet, there are many standards for developing NFT smart contracts, such as ERC-721 \1155 \998, each of which has its own characteristics, but their characteristics are expanded on the basic properties. We choose the ERC-721 standard to develop NFT smart contracts, in the metadata storage section, there is tokenUrl equivalent to the unique id of the substance. links to files stored on top of IPFS or other services, but are not limited to links, but can also be other content.

The entire contract needs to have the following binding features. a) NFT holder. That is, msg.sender(owner) and tokenId are in a one-to-many relationship, representing that a person can have multiple NFTs. b) tokenId and tokenUrl have a one-to-one relationship, representing a unique id in the chain for each copy of data, while tokenUrl is not required to be unique, but on the caller side, tokenUrl is usually set to be unique, even if it is not unique, it does not matter, and in case of conflict, the smaller the tokenId, the earlier it was set in the first place. c) After writing data to the chain, the NFT holder is able to obtain the unique id of the chain for the NFT and can subsequently perform a series of read and write operations based on the id.

### Liquidity Layerzero cross-chain model based on super-contract pairs

**Fig 2:**
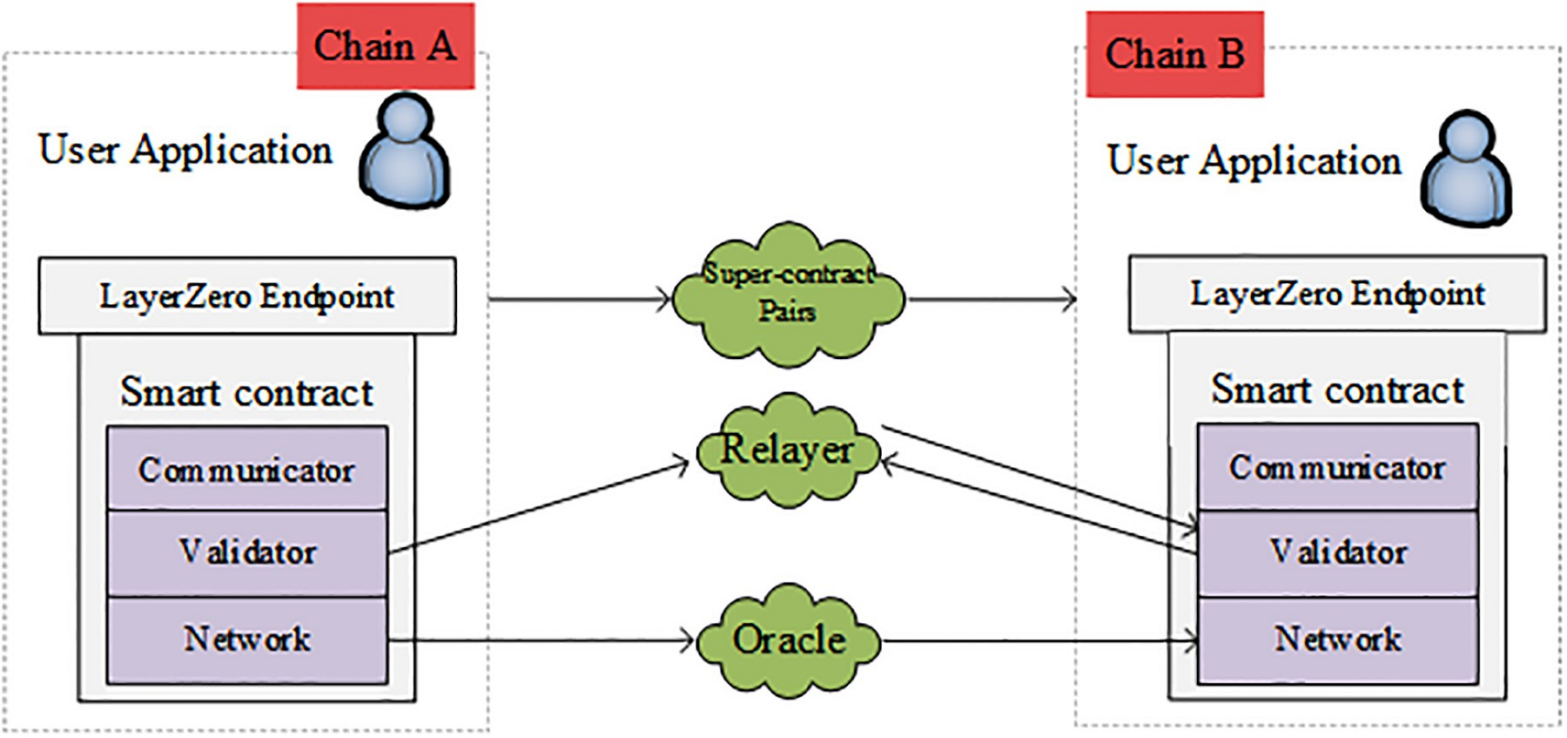
Cross-chain model diagram of liquid Layerzero based on super-contract pairs.

This section introduces the cross-chain model of liquidity Layerzero based on super-contract pairs and analyzes the concept of super-contract pairs and the principle of the liquidity Layerzero cross-chain model. The cross-chain model diagram of liquid Layerzero based on super-contract pairs is shown in Fig 2.

Layerzero, a super-contract pair-based liquidity, is a chain-wide interoperability protocol that builds a new ultra-light node model to provide a secure and reliable infrastructure for various cross-chain protocols. Equivalent to running a chain-wide ultralight node, Layerzero relies on Oracle and Relayer to transfer information between Layerzero Endpoint on different chains, passing block headers (block headers contain transaction information, message m, etc.) through Oracle. Relayer transmits proof of transaction (Proof information), verifies each other to ensure security, and collaborates with super-contract pairs to build a free-flowing cross-chain. In summary, Layerzero, liquidity based on super-contract pairs, enables a direct interoperability model for free liquidity between any blockchain.

The liquidity Layerzero cross-chain model based on Super-contract pairs contains four core components: Super-contract pairs, Endpoint, Oracle, and Relayer.

#### Super-contract pairs

**Fig 3:**
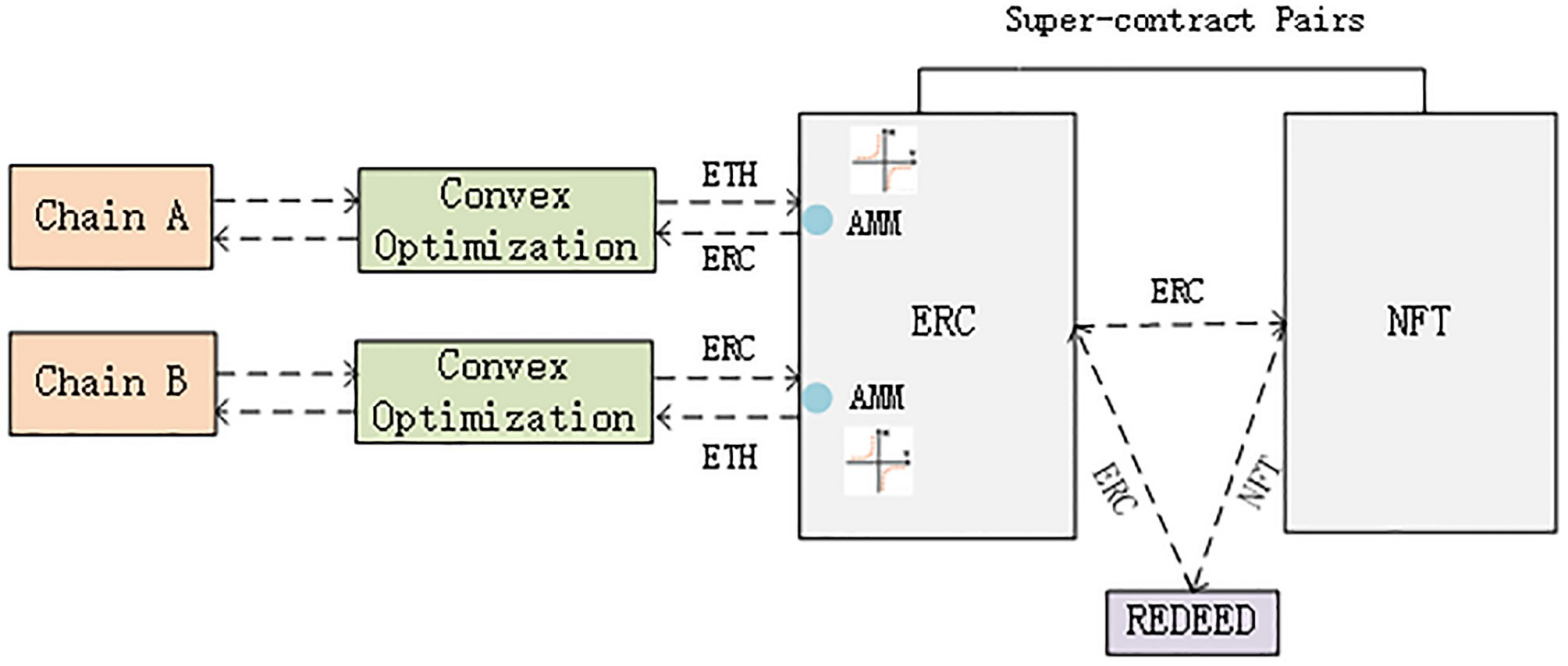
The conceptual diagram of the liquidity super-contract pairs.

The ERC (Ethereum Request for Comment) pass-through standard is a specification for creating pass-throughs through Ethereum. According to the ERC specification, a smart contract can be written to create “fungible passes”. Deploying a super-contract pair consisting of an ERC transaction contract and an ERC super-object contract, on the one hand, allows NFT to be decentralized across chains, making it truly mobile and instantaneous; on the other hand, it allows NFT to conduct “fragmented” transactions, lowering the threshold for NFT to cross chains. The dynamics and supply of ERC are managed by the bonding curve as AMM (Automated Market Maker) and have instant liquidity, and the contract automatically adjusts the liquidity trading credit according to the circulating supply. The conceptual diagram of the liquidity super-contract pairs is shown in Fig 3.

NFT is stateful, programmable, and networked. A graph is formed in which items, markets, and social are wrapped in it. Each piece of information becomes its own micro-economy with native incentives for investment builders to develop spaces and systems for the ecology of items. This is a better way to implement a scenario of liquid cross-chain transactions.

*AMM liquidity trading mathematical model*. A trading pair is based on two Tokens, let’s assume they are X and Y. Then x and y are the number of tokens X and Y have as reserves in this trading pair. This ratio of pairs is determined based on the number of X and Y in the pair. The k iny*x = k is certain during the whole trading process. It is possible to keep the two coins to be traded at a corresponding price and quantity all the time. Set the trading formula as shown below.

$$\begin{array}{c} x*y=(x+\delta x)*(y-\delta y) \end{array}$$ (2)

Where *δx* represents the value-added coins and *δy* represents the sold coins, set *α* = *δx*/*x* and *β* = *δy*/*y*. The equations that can be derived are shown below.

$$\begin{array}{c} x+\Delta x=\frac{xy}{y-\Delta y}=\frac{1}{1-\beta }x \end{array}$$ (3)

$$\begin{array}{c} y-\Delta y=(1-\beta )y \end{array}$$ (4)

$$\begin{array}{c} \Delta x=\frac{\beta }{1-\beta }x \end{array}$$ (5)

$$\begin{array}{c} \Delta y=\frac{\alpha }{1+\alpha }y \end{array}$$ (6)

When the *k* of the model is not constant but will have a slow growth with the transaction, it shows the liquidity share calculation and updates to achieve the mathematical model of increasing liquidity across the chain of transactions, as shown in the following equation.

$$\begin{array}{c} \left(e,t,l)\to (e^{\prime },t^{\prime },l^{\prime }\right) \end{array}$$ (7)

where *e* represents the number of ether, *t* represents the number of transactions to another coin, and *l* represents the number of increased liquidity. *e*′, *t*′, *l*′ represent the values after the state has changed, respectively, and are all at rest, that is, their values change only after changing from one state to the next, and let *α* = *δe*/*e*, that is

$$\begin{array}{c} e^{\prime }=\left(1+\alpha \right)e \end{array}$$ (8)

$$\begin{array}{c} t^{\prime }=\left(1+\alpha \right)t \end{array}$$ (9)

$$\begin{array}{c} l^{\prime }=\left(1+\alpha \right)l \end{array}$$ (10)

where the ratio of e:t:l is fixed, i.e.

$$\begin{array}{c} e:t:l=e^{\prime }:t^{\prime }:l^{\prime } \end{array}$$ (11)

#### Endpoint

Liquidity Layerzero deploys an outlet (i.e. a series of smart contracts that can handle logic) on both chains to each other to interact with the other chain, called an “Endpoint”. The user application calls send to transfer messages and set up its own UA-configured deployed contract. It is also understood that Endpoint will run a “super light node”. The core components of the Endpoint are Communicator, Validator, and Network, whose function is to notify the super-contract pairs, Oracle, and Relayer to get specific information and receive messages from them.

#### Oracle

**Fig 4:**
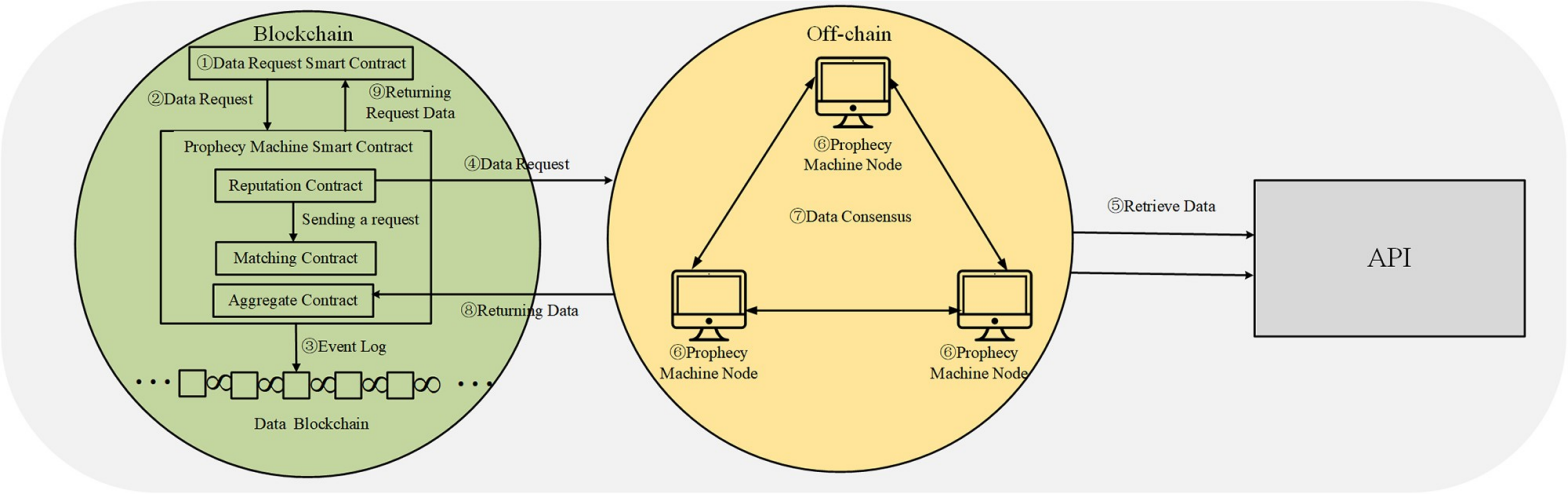
Prophecy machine working schematic.

The closed nature of the blockchain system makes it not only consumes a lot of resources in obtaining outside information but also the authenticity difficult to guarantee. The emergence of the prophecy machine solves this problem, which introduces credible external data to the blockchain system and provides the necessary conditions for data sharing and exchange between smart contracts in the system and external systems. This data is necessary for the smart contract to run when the conditions are met and can be any data related to the smart contract: temperature, payment completion, price, etc. Also, the prophecy machine itself is a smart contract that allows the blockchain to connect to any existing API and allows smart contracts to interact with other blockchains. The prophecy machine is tamper-proof, service-stable, auditable, and powered by incentives to run. The schematic diagram of the operation of the prophecy machine is shown in Fig 4.

*On-chain mechanism*. There are four types of smart contracts included in the blockchain on-chain mechanism, namely data request a smart contract, reputation contract, matching contract, and aggregation contract.

- Data Request Smart Contract. Also called user contracts, they are the demanders of data.
- Reputation contracts. It can be benchmarked against the intangible asset “goodwill”, which refers to the valuation of reputation, but the subject of reputation covers all organizational forms such as natural persons and merchants, and all content forms such as novels, music, movies, and dramas. At the same time, the reputation contract will check the historical service level of the prophecy machine service provider, verify its authenticity and historical performance, and eliminate the prophecy machine nodes with a poor reputation or low reliability.
- Matching contract. The data request from the request contract is sent to the node and the bid from the node is accepted. Then the order-matching contract selects the appropriate number and type of prophecy machines to complete the task.
- Aggregation Contract. Get all the data from the chosen prognosticator, validate and aggregate the data, and finally produce accurate results. The aggregation contract can validate data from a single data source or from multiple data sources. In addition, it can aggregate data from multiple data sources.

At work, the data request smart contract sends a data request when it has a data demand. After the reputation contract receives the confirmation from the user contract, the predictive machine submits the data request. Then the data node receives the request, makes a data query, and submits the query result to the aggregation contract, which aggregates the data, and submits the final query result to the user contract, finally completing the user’s data query request.

*Off-chain consensus algorithm*. The (t,n) threshold signature technique is used to achieve off-chain consensus in the distributed prophecy machine network. The (t,n) threshold signature technique is to have a public-private key pair in a signature population consisting of n participants, and this private key is sliced into many pieces in a certain way and distributed to all participants in the population. When any number of participants in the group greater than or equal to t sign the same data using their respective private key fragments, a complete and valid signature is generated. where t is the threshold value and also the minimum number of participants with respect to generating a legitimate signature, when the number of signatories is less than t, then no valid signature can be generated.

The off-chain consensus process consists of 4 phases: key generation, signature generation, signature verification, and prophet reward mechanism. Through the threshold signature technique, prophet nodes in a distributed prophet network interact with each other off-chain. When prophecy machine nodes generate partial signatures separately for a total of one value, they can aggregate into a complete key signature and approve the data transmission to send the consensus data to the prophecy machine-wise contract on the blockchain.

Assuming that there are *f* < *n*/3 prophecy machines with a threshold *t* = *f* + 1, the problematic nodes may have “empty pay” behavior or other dishonest operations, such as illegal signatures. The complete off-chain consensus process consists of the following four stages.

(1) Key generation. Execute the distributed key generation protocol, outputting the global public key *Y* and each propagator node *O*, each all key shares *s*_*i*_ and public key shares *Y*_*i*_.
(2) Signature generation. Each propagator node generates partial signatures *δ*_*i*_ by its own key share *s*_*i*_ and then aggregates them to obtain verifiable full signatures *δ*.
(3) Signature verification. After the aggregated prophecy machine submits data D and signature *δ*, the prophecy machine-wise contract verifies the signature based on the public parameters and global public key *Y*.
(4) Prophecy Machine Reward. The prophecy machine smart contract rewards the prophecy machine nodes that have truly acquired the data to generate the signature without annoying your threshold according to the data submitted by each prophecy machine node.

Suppose the set of propagator nodes participating in consensus is *O* = *O*_1_, *O*_2_, *O*_3_, …, *O*_*n*_, with a total of *n* propagator nodes and a threshold value of *t*. The security parameter *k* is selected and a cyclic group *G* of order *q* is chosen whose discrete logarithm problem is intractable with finite field *Z*_*q*_ with a number of elements *q* and eigenvalues *p*. *p*, *q* are large prime numbers and *g* is a generating element of *G*. Choose secure hash function $H_{1}:\{0,1{\}}^{*}\to Z_{q}^{*}$, $H_{2}:\{0,1{\}}^{*}\to Z_{q}^{*}$. The overt parameters are *k*, *p*, *q*, *g*, *G*, *H*_1_, and *H*_2_. In addition, we denote by *x* ← *S* the selection of a random number *x* from *S*.

#### Relayer

Relayer is used in the cross-chain model to obtain proof of transaction events. The Layerzero cross-chain model is based on ultralight nodes linking full chains, and ultralight node *V* connects multiple full nodes *P*, at least one of which is a non-evil full node. *V* requests *P* to verify whether a transaction really exists on the chain, *P* returns the relevant proof, and V makes a judgment based on the result returned by *P*.

The first step of the determination is to identify the non-evil full node, and the second step is to verify whether a transaction really exists on the chain based on the data refig 4 by that node.

### Dynamic trading node creditworthiness evaluation model

#### Dynamic evaluation model

**Fig 5:**
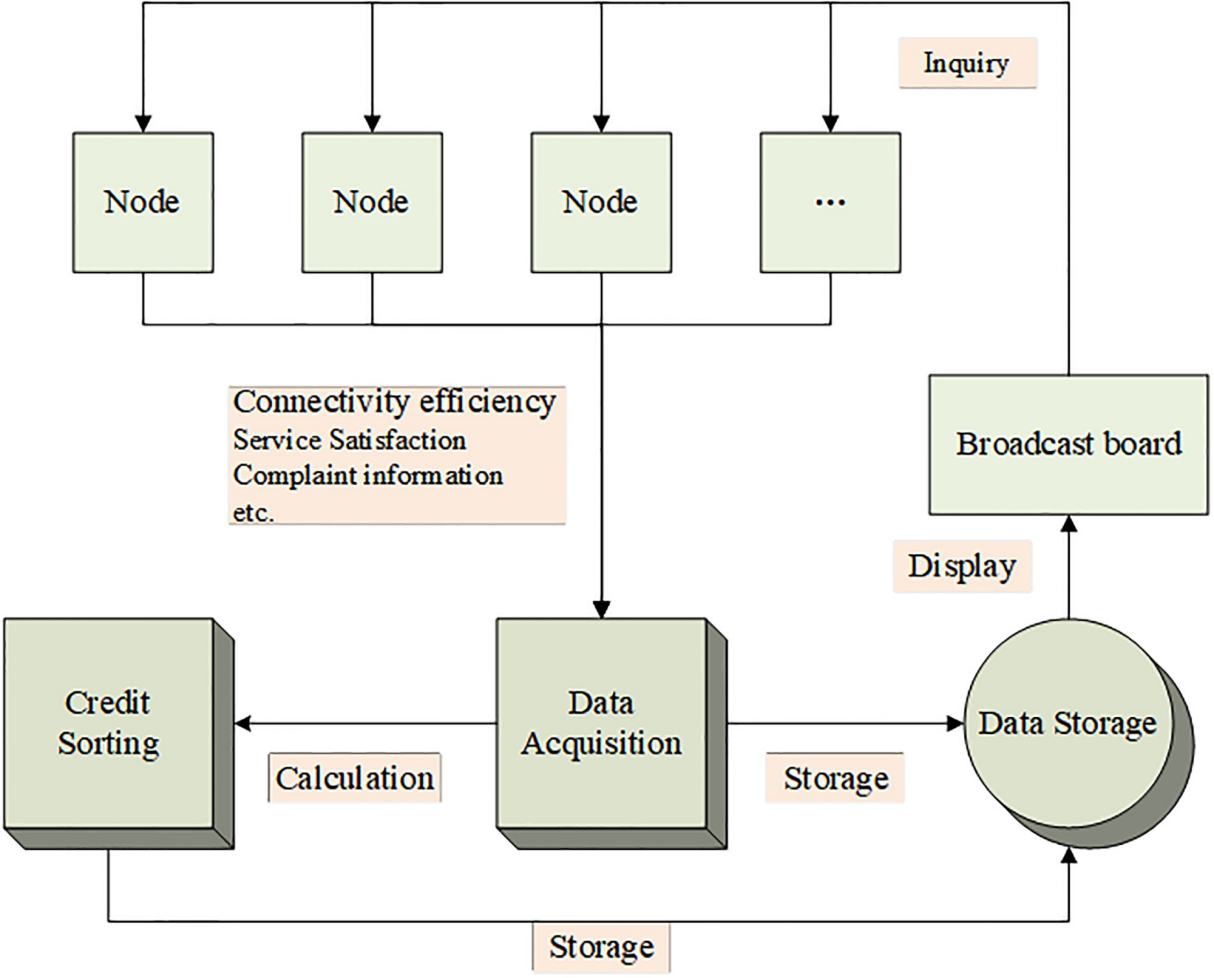
Working schematic of node evaluation model.

The liquidity cross-chain transaction node creditworthiness evaluation model consists of a broadcast version, transaction node module, data collection module, data storage module, and credit ranking module, and the working principle is shown in Fig 5 [28–31].

The broadcast board is a module for displaying voting content, transaction node evaluation content, and announcement information. The voting content contains the information of the trading node voting, the signature Fig 5 of the voter, and the signature of the broadcast board; the evaluation process contains the evaluation content and the evaluation result; the announcement information includes the addresses of the trading nodes participating in the voting and the voting start and end times. The trading node module is used for filling in voting information and verification of trading nodes [32]. It includes a node login verification module and an information filling module. The data collection module is used to collect transaction node evaluation information and historical transaction evaluation information. The data storage module is used to store node information and credit ranking information [33–35].

**Fig 6:**
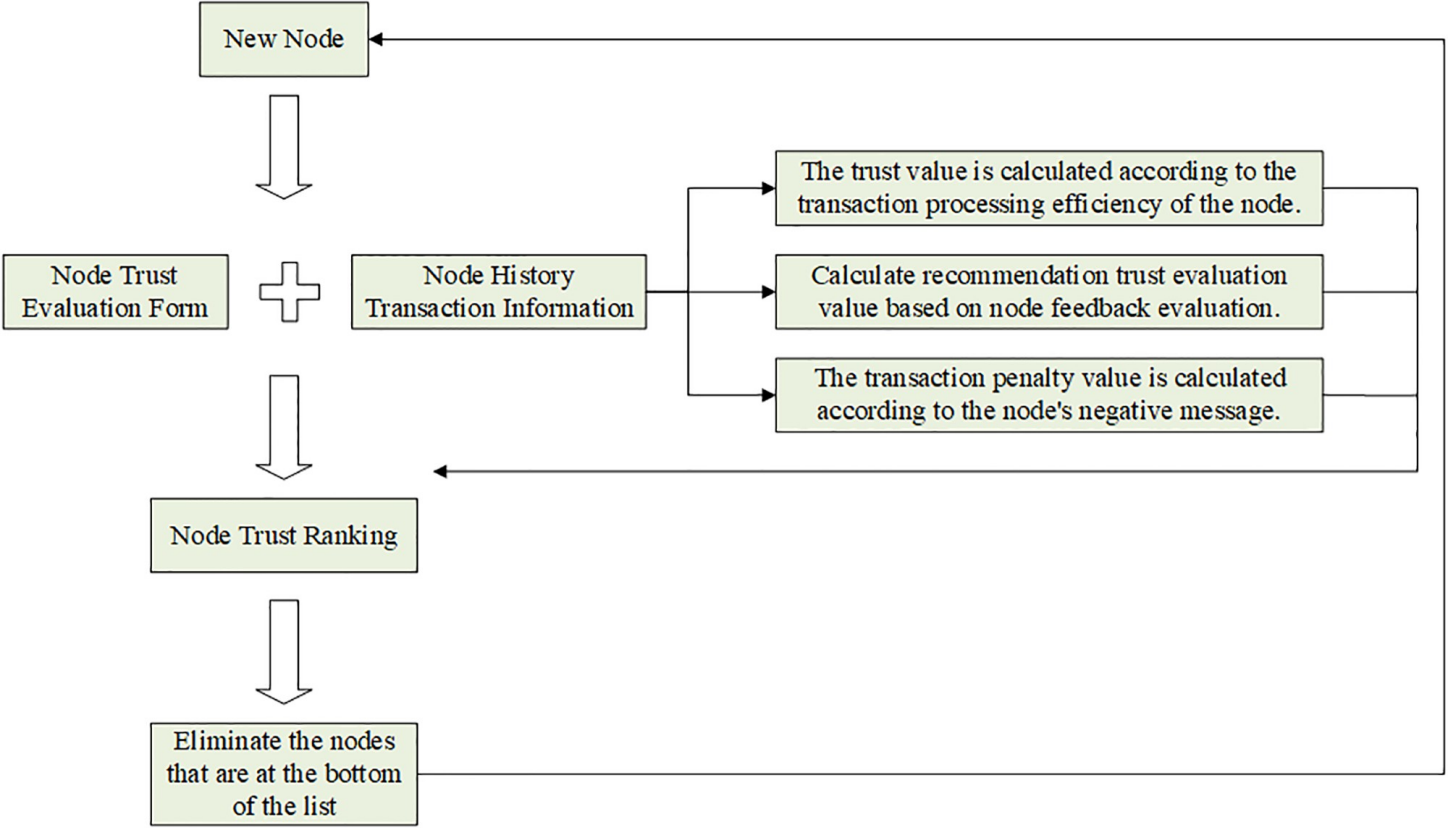
Evaluation flow chart.

In this model, within a specified time, the system evaluates the trust degree of existing nodes; the system will send evaluation signals to the existing nodes and broadcast the system public key and address; each node receives the signal, encrypts it using the system public key and encrypts the signature using its own private key; the system will collect the encrypted data of each node and decrypt it using the system private key, and finally form the trust relationship graph and broadcast it. If the system does not receive any objection information within the specified time, it proceeds to the next step of the plan, and if there is, it re-votes. The system automatically collects the historical transaction information of each node ranks the creditworthiness, and announces the results and the calculation process, if the system does not receive any objection information within the specified time, it automatically eliminates the nodes ranked at the bottom according to the process. The flow chart of transaction node evaluation is shown in Fig 6.

Among them, the user feedback evaluation in the transaction node’s historical transaction information refers to the user’s evaluation of the transaction after its final completion, with the score value ranging from 1–5 from low to high. The transaction node processing efficiency refers to the time required by users from initiating a transaction to final confirmation of the transaction, and the negative transaction information includes transactions that are not completed and transactions with fraud and other behaviors.

#### Node trust model

The set of all participants in a cross-chain is called the set of nodes, denoted as *E*. The trust degree of a node, as a notary, is a quantitative representation of the degree of trust of the relevant nodes and is a cumulative evaluation reflecting the behavior of nodes in the long-term operation of a cross-chain. To cope with the trust requirements of cross-chain scenarios, the trust degree values of nodes are distributed in the interval of real numbers. The request to evaluate the trust degree *T* of node *i* to node *j* consists of the direct trust value *DT*(*i*, *j*), the recommended trust value *RT*(*i*, *j*), and the negative penalty value *PT*(*i*, *j*), which is calculated as follows.

$$\begin{array}{c} T=\alpha DT(i,j)+\beta RT(i,j)-\gamma PT(i,j) \end{array}$$ (12)

The direct trust *DT*(*i*, *j*) is the trust evaluation of node *j* by the requesting evaluation node *i* ∈ *E* based on the historical interaction experience with the evaluated node *j* ∈ *E* [19]. If requesting node *i* and node *j* have directly occurred in a period or *N* transaction transmissions, the satisfaction of the *n*th transaction transmission is *S*(*i*, *j*) ∈ [0, 1], which takes the values shown in the table. The thing impact factor of the *n*th event is *IF*(*j*, *n*), then the direct trust value of requesting node *i* to node *j* is

$$\begin{array}{c} DT\left(i,j\right)=\frac{\sum_{n=1}^{N}S\left(j,n\right)IF\left(j,n\right)\delta_{k}}{\sum_{n=1}^{N}IF\left(j,n\right)\delta_{k}} \end{array}$$ (13)

where *IF*(*j*, *n*) is the transaction impact factor of the nth interaction, *IF*(*j*, *n*) ∈ (0, 1); *δ*_*k*_ is the time decay factor corresponding to a period $\delta_{k}=e^{-\frac{1}{k}}$, *k* ∈ *T*_*k*_, which is a monotonically increasing function, the larger the *IF*(*j*, *n*) is from the current transaction time; *T*_*k*_ is the set of transaction periods. The node evaluation satisfaction values are taken as shown in Table 3.

**Table 3:** Node evaluation satisfaction values.

Service Quality	Satisfaction	Service Description
Better	1	The service node provided high-quality service with a very good deal.
Good	0.75	The service node provides the correct service and lesser services have delays.
General	0.5	The service node provides the correct service, but the service is somewhat delayed or degraded.
Bad	0.25	The service nodes provide some of the correct services and most of the services are somewhat delayed or degraded.
Worse	0	The service node rejects a reasonable response to a service request or provides an error message.

Direct trust exists only between nodes that have already had cross-chain transactions, however, evaluation nodes often encounter uninteracted nodes with no direct experience to refer to, and then they can only rely on recommendations from other nodes. The recommended trust value *RT*(*i*, *j*) is the trust judgment of a node formed by the evaluation node *i* ∈ *E* based on the trust evaluation of node *j* ∈ *E* provided by a third-party node *k* ∈ *E*. When node *i* evaluates node *j*, its interaction history with node *j* alone may not be objective enough, and the trust information of other entities recommending the evaluated node is still needed, especially if node *i* has never interacted with node *j*. The trust value *RT*(*i*, *j*) is

$$\begin{array}{c} RT\left(i,j\right)=\frac{\Sigma_{m\in G}DT\left(m,j\right)Ht\left(i,m\right)\text{Sim}\left(i,m\right)}{\Sigma_{m\in G}DT\left(m,j\right)} \end{array}$$ (14)

*G* is the set of recommenders; *DT*(*m*, *j*) is the direct trust value of the recommended node *m* to node *j*; *Ht*(*i*, *m*) is the recommendation trustworthiness of the recommended node *m* according to node *i*, which indicates the recommendation ability of the recommended node *m*; *Sim*(*i*, *m*) is the similarity between the recommended node *m* and the evaluation of node *i*’s trust evaluation of node *j*. The higher the similarity, the more node *i* and node *m* agree on each other the more consistent node *i* and node *m*’s perceptions of other nodes in the network. Can the recommending node provide a trustworthy recommendation? In *G*, the node with higher similarity is generally selected as the recommendation node, and the appropriate recommendation node can be selected by setting a similarity threshold ∂ ∈ [0, 1] such that *Sim*(*i*, *m*) ≥ ∂.

The degree of credibility is calculated as follows.

$$\begin{array}{c} Ht\left(i,m\right)=\frac{S_{t}\left(i,m\right)}{S_{t}\left(i,m\right)+F_{t}\left(i,m\right)} \end{array}$$ (15)

*S*_*t*_(*i*, *m*) denotes the number of satisfactory recommendations provided by recommendation node *m* among all transaction histories of requesting node *i*; *F*_*t*_(*i*, *m*) is the number of unsatisfactory recommendations provided.

The degree of similarity between two nodes is portrayed by the cosine similarity function, and the similarity is calculated as follows.

$$\begin{array}{c} \text{Sim}\left(i,m\right)=\frac{\sum_{m}DT\left(i,m\right)DT\left(j,m\right)}{\sqrt{\sum_{m}DT^{2}\left(i,m\right)}\sqrt{\sum_{m}DT^{2}\left(j,m\right)}} \end{array}$$ (16)

*S*_*t*_(*i*, *m*) denotes the number of satisfactory recommendations provided by recommendation node *m* among all transaction histories of requesting node *i*; *F*_*t*_(*i*, *m*) is the number of unsatisfactory recommendations provided.

The degree of similarity between two nodes is portrayed by the cosine similarity function, and the similarity is calculated as follows.

*m* is the node that has traded with both node *i* and node *j*.

The negative transaction penalty value *RT*(*i*, *j*) can be understood as the price paid by a node for causing trust fluctuations after using its trustworthiness to make false transactions, and the purpose of introducing the penalty value is to impose a certain penalty effect on nodes whose behavior changes dynamically by assigning appropriate weights to make the node’s trustworthiness fall more quickly, to achieve suppression of dynamic changes in node behavior.

$$\begin{array}{c} RT\left(i,j\right)=\frac{\sum_{k\in T_{k}}\delta_{k}\;\text{max}(0,RT^{k}\left(i,j\right)-DT^{k}\left(i,j\right)}{\sum_{k\in T_{k}}\delta_{k}} \end{array}$$ (17)

*RT*^*k*^(*i*, *j*) is the recommended trust value of node *j* that can be obtained by requesting node *i* by pooling all recommended nodes in time period *k*; *DT*^*k*^(*i*, *j*) is the direct trust value of requesting node *i* to node *j* in time period *k*; *δ*_*k*_ is the time decay factor corresponding to time period *k*.

## Experiment analysis and results

In this section, we first build the experimental environment for this study, and then we build the underlying blockchain framework in the environment and launch multiple application chains on different blockchain frameworks, such as Ethernet and Optimism to configure the system and confirm the software configuration [36–38]. At the same time, two cross-chain models are deployed respectively, one is based on the basic Layerzero cross-chain model, and the other is based on the super-contract pairs with the liquidity of the Layerzero cross-chain model, and finally, the final analysis is summarized by comparing the model’s time-consumption, the smart contract’s robustness, and the cross-chain model’s radius of convergence of the two cross-chain models in the final analysis of the performance indexes.

### Experimental analysis

In our experiments, we use three performance metrics, namely the time consumption of the cross-chain model, the robustness of the smart contract, and the radius of convergence of the cross-chain model, as the basis for the experimental validation of the strength of liquidity. The experimental results are compared for two sets of models, one basic Layerzero cross-chain model and the other liquid Layerzero cross-chain model based on super contract pairs.

Before cross-chaining, the information of cross-chaining is firstly unified identity NFT id, followed by two groups of cross-chaining models respectively. In order to verify the correctness and adaptability of the model, the data contains 10 groups of experimental data mixed with different file categories, and the experimental data are configuration data, account data, block data, transaction data, entity data, contract data, and data containing large files, confidential and sensitive data, and redundant and repetitive data, and the file sizes of each group are 50B, 100B, 150B, 200B, 250B, 300B, 350B, 400B, 450B, and 500B.

### Test performance

In blockchain cross-chain transactions, the efficiency of cross-chain technology can be weighed in terms of its relevant performance metrics. Therefore, in this section, three important characteristic indicators in cross-chain transactions are analyzed to enhance the liquidity cross-chain model. The three important characteristic metrics in the liquidity cross-chain model are as follows.

Performance measure 1: Time-consumption of liquidity cross-chain model
Performance measure 2: Robustness of liquidity cross-chain smart contracts
Performance measure 3: Radius of convergence of liquidity cross-chain model

Next, experiments are conducted to verify the time-consumption, robustness, and radius of convergence of the model’s characteristic metrics, with one group experimenting by deploying the original Layerzero cross-chain model, and the other group experimenting by deploying the liquidity replacement cross-chain contract based on super-contract pairs.

**Performance measure 1**. In the blockchain cross-chain model, the time spent in the process of transferring or circulating value by some technical means is called cross-chain time-consuming. Tables 4 and 5 represent the time required to perform cross-chain experiments for the base Layerzero cross-chain model and the proposed liquidity cross-chain mode. By comparing Tables 4 and 5, it can be seen that the Table 4 scheme takes longer time than the Table 5 scheme, regardless of the file types transmitted across the chain. This indicates that the time-consuming nature of cross-chaining is very little affected and within a certain controllable range, after the NFT ID is unified for different file types, and at the same time, the cross-chaining authentication time and data memory can be greatly reduced, which is more convenient for cross-chaining transmission.

**Table 4:** Base Layerzero cross-chain model timeliness.

*Group*\*Bytes*	50	100	150	200	250	300	350	400	450	500
Group1	210	219	235	257	291	321	377	412	471	521
Group2	213	220	233	255	296	327	373	427	476	517
Group3	209	224	237	259	293	331	381	431	462	530
Group4	215	218	239	260	287	335	380	436	468	531
Group5	211	226	242	262	289	326	383	421	474	548
Group6	208	227	238	261	294	324	386	435	482	519
Group7	214	222	244	258	301	339	379	425	488	526
Group8	209	223	241	266	295	345	387	441	490	527
Group9	211	218	237	269	303	341	390	447	477	516
Group10	212	226	243	270	305	348	392	437	481	538
Average	211.1	224.2	243.3	269.8	303.1	347.7	392.3	444.4	487.1	530.2

**Table 5:** Liquidity cross-chain modeling timeliness.

*Group*\*Bytes*	50	100	150	200	250	300	350	400	450	500
Group1	180	184	188	190	196	201	208	215	220	229
Group2	177	180	185	194	198	203	210	214	221	227
Group3	173	175	185	195	199	205	210	216	222	227
Group4	172	178	187	192	195	201	212	216	221	226
Group5	170	179	186	193	199	204	208	214	222	228
Group6	171	176	187	194	201	205	209	213	221	227
Group7	176	182	189	196	202	206	212	217	223	228
Group8	175	184	190	195	201	206	210	215	223	229
Group9	171	179	187	193	198	205	210	216	225	229
Group10	178	185	190	196	200	207	212	218	226	229
Average	173.1	182.1	189.5	195.8	201.2	207.3	211.3	216.7	225.5	228.9

**Fig 7:**
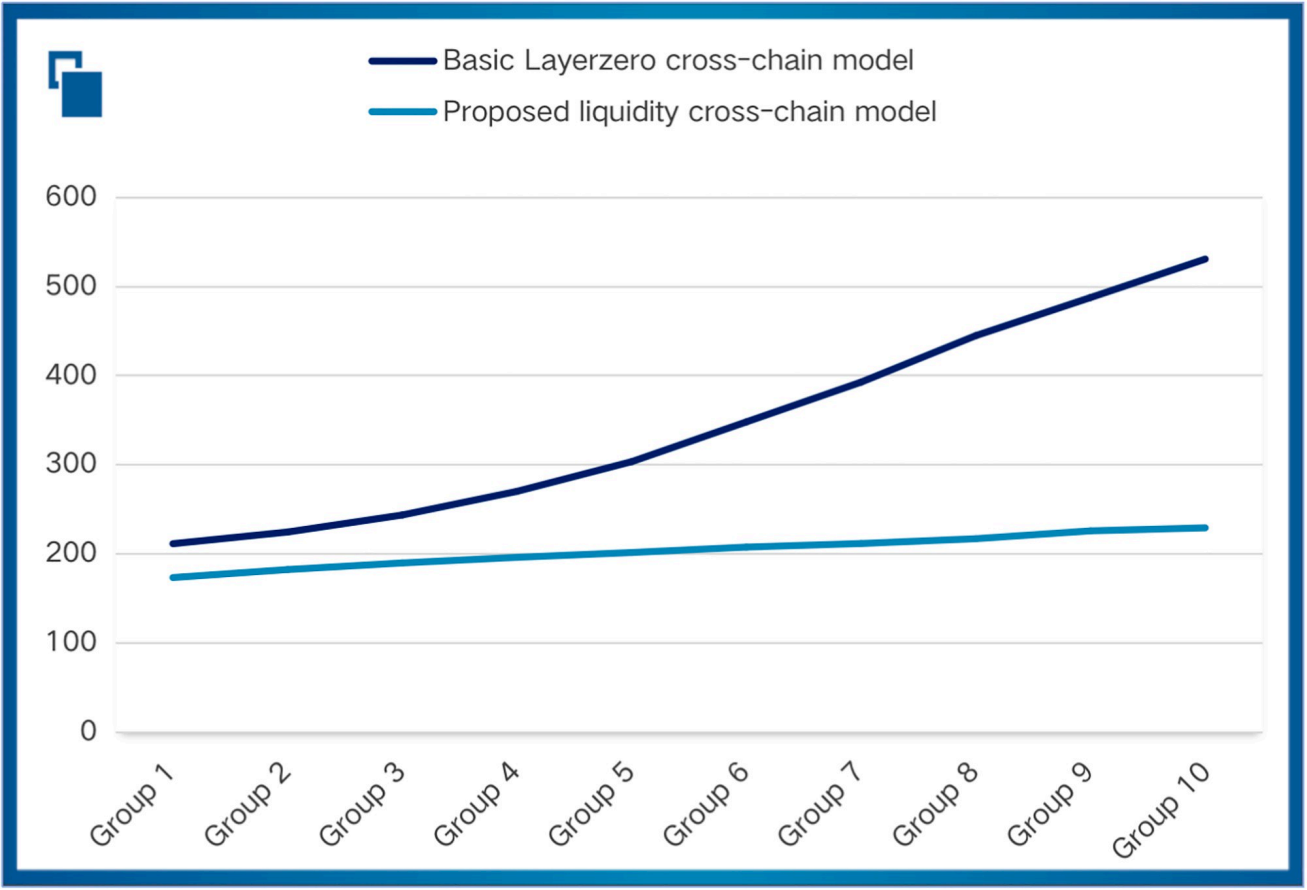
Timeliness comparison chart.

We visualized the two sets of experimental data as shown in Fig 7. Where the horizontal axis indicates the cross-chain group and the vertical axis indicates the number of bytes of cross-chain data and the average time required to cross the chain (ms). The dark blue curve represents the basic Layerzero cross-chain model across the chain, and the light blue curve represents the proposed liquidity cross-chain model. It can be concluded that the proposed liquidity cross-chain model in this paper can improve the timeliness by 18% when the number of bytes is 50 and 56.8% when the number of bytes is 500. For a given number of bytes, the base model cross-chaining takes more time than the proposed liquidity cross-chaining model. It is also more noteworthy that as the number of bytes increases, the time required for the base cross-chain model multiplies by a substantial upward trend, while the proposed mobility cross-chain model can basically maintain a smooth trend. This suggests that the liquidity cross-chain model is more stable compared to the basic cross-chain model in the future large-scale cross-chain environment.

**Fig 8:**
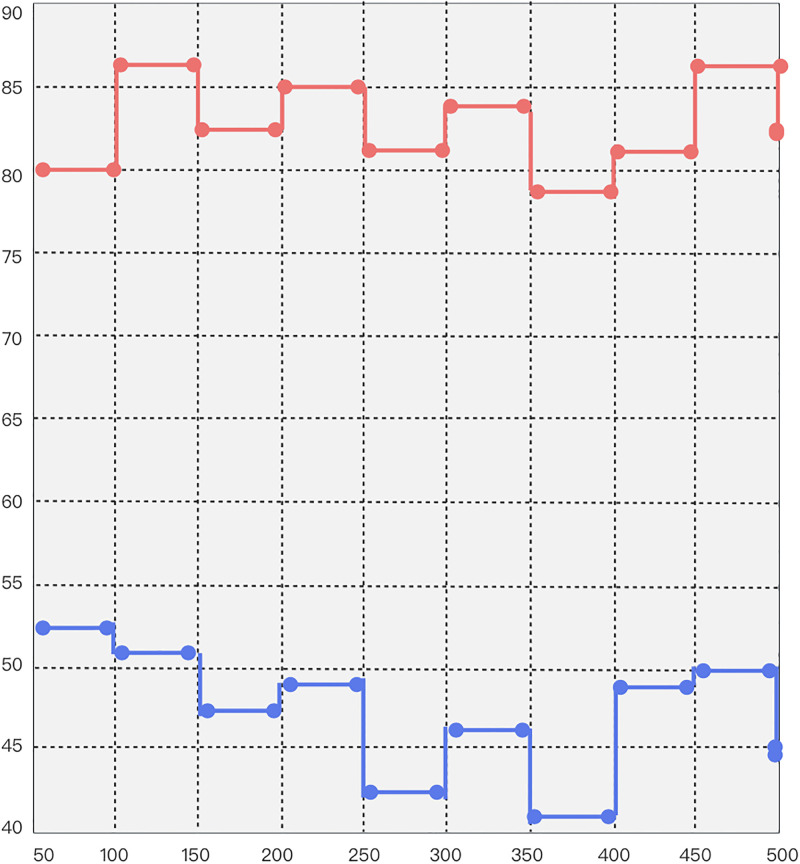
Robustness comparison chart.

**Performance measure 2**. In the blockchain cross-chain model, the nodes of the blockchain system model are used as the point set, the block states as the line set, and the blockchain performance as the surface for defining the robustness parameters of the consensus algorithm in the liquidity cross-chain model, followed by categorizing and analyzing the robustness parameters of the consensus algorithm of the liquidity cross-chain model and finally establishing the robust nodes that ensure the best operation of the liquidity cross-chain model to constitute the initial design set. As shown in Table 6 and Fig 8, the average robustness metrics for 10 iterations of different-sized data sets in mobility cross-chain interoperation are displayed. The robustness of the current model is much higher than that of the comparison group, with a minimum improvement of 50.9% and a maximum improvement of 92.7%. This indicates that the liquidity cross-chain model cross-chain rate variation adjusts accordingly to the cross-chain demand dataset and can resist a large number of errors, applauds, and overloads. Further, it can be concluded that the current model is very resistant to data trading in the same situation.

**Table 6:** Comparison of the robustness of the liquidity cross-chain model and the basic model.

Bytes	Liquidity cross-chain model	Basic model
50	80	53
100	86	51
150	83	47
200	85	49
250	81	43
300	84	46
350	79	41
400	81	49
450	86	50
500	82	45

**Performance measure 3**. As cross-chain transactions continue to increase, the cross-chain model must continually reach a stable operating point to ensure that cross-chain transactions run fluidly. When a cross-chain transaction reaches a proximity point, the radius of convergence is used to ensure the security and stability of the transaction, so the smaller the radius of convergence proves that the model is more secure and stable. As shown in Table 7, shows the radius of convergence in cross-chain model interoperation when different-sized data sets converge to secure stability after iterations. By observing the number of iterations of the model across the chain, it can be proved that the current technology ensures security and stability only when the number of bytes is less than 150, whereas the proposed mobility cross-chain model can achieve stable operation all the time. Also, the radius of convergence of the proposed model is reduced by at least 25% and up to 68.6%. Therefore, the proposed model is very secure and has strong risk management.

**Table 7:** Comparison of radius of convergence of the liquidity cross-chain model and the basis model.

Bytes	Liquidity cross-chain model	Basic model
50	10	15
100	12	16
150	11	15
200	12	18
250	14	22
300	15	28
350	14	39
400	13	40
450	15	46
500	16	51

## Conclusion

### Model comparisons

In this paper, we propose a liquidity Layerzero cross-chain model framework based on super-contract pairs. Firstly, it unifies the distributed autonomous cross-chain NFT identity ID to facilitate easier access to the cross-chain service framework. Secondly, it combines the ERC-based super contract pair model with the AMM bonding curve management to realize the free liquidity transfer of digital assets and the free interoperability of information, to achieve the effect of instant liquidity. After that, the dynamic transaction node evaluation model and node trust model are designed to ensure the processing efficiency of the transaction nodes and increase the security of the cross-chain model.

The experiments are conducted by comparing two cross-chain models, the proposed model and the state-of-the-art Layerzero cross-chain model. Based on three characteristic metrics in the cross-chain model, which are timeliness of the model, robustness of the smart contract, and radius of convergence of the cross-chain model. It is concluded that the proposed liquidity cross-chain model improves the timeliness by at least 18%. In contrast, the efficiency of the model is not affected much and is within a specific controllable range with the increase of time and throughput, the robustness increases by at least 50.9%, and the radius of convergence decreases by at least 25%. In summary, the liquidity Layerzero cross-chain model based on super contract pairs is more stable, efficient, and secure.

### Limitations and future work

In this study, intelligent consensus mechanisms such as throughput and incentives in the model for the service chain need to be further improved [39, 40]. Meanwhile, as an emerging technology in the digital asset sector, the circular liquidity cross-chain model has a broader outlook. With the continuous development of cryptocurrencies and liquidity cross-chain, the cross-chain model needs to further improve the privacy and security features to provide more options for digital asset holders. Multi-chain attributes and circular flow cross-chain models complement each other to provide users with more cross-chain transaction opportunities and promote the further development of the digital currency market.

In the future, the liquidity cross-chain model will serve as a foundation to assist the circular liquidity cross-chain model to play an important role in the digital currency market.

## Supporting information

S1 Dataset (ZIP)
